# Precise engineering of Gemcitabine prodrug cocktails into single polymeric nanoparticles delivery for metastatic thyroid cancer cells

**DOI:** 10.1080/10717544.2020.1790693

**Published:** 2020-07-16

**Authors:** Chenggong Liu, Qiongmei Han, Hua Liu, Cuirong Zhu, Wei Gui, Xiaodong Yang, Wansen Li

**Affiliations:** a Department of General Practice, Zhumadian City Central Hospital, Zhumadian, China; b Department of Endocrinology, Yankuang New Journey General Hospital, Jining, Shandong, China; c Excellent Ward, Zhumadian City Central Hospital, Zhumadian, China; d Department of Gynaecology and Obstetrics, Zhumadian Women and Children's Health Hospital, Zhumadian, China; e Department of Pharmacology Department, Zhumadian First People's Hospital, Zhumadian, China

**Keywords:** Gemcitabine prodrug, single polymeric nanoparticles, thyroid cancer, apoptosis

## Abstract

GLOBOCAN estimates 36 types of cancers in 185 countries based on the incidence, mortality, and prevalence in the year 2019. Nowadays, chemotherapy is the most widely used cancer treatment among immune, radio, hormone, and gene therapies. Here, we describe a very simple yet cost-effective approach that synergistically combines drug reconstitution, supramolecular nano-assembly, and tumor-specific targeting to address the multiple challenges posed by the delivery of the chemotherapeutic Gemcitabine (GEM) drug. The GEM prodrugs were gifted to impulsively self-assemble into excellent steady nanoparticles size on covalent conjugation of linoleic acid hydrophobic through amide group with ∼100 nm. Newly synthesized GEM-NPs morphology was confirmed by various electron microscopic techniques. After successful synthesis, we have evaluated the anticancer property of GEM and GEM-NPs against B-CPAP (papillary thyroid carcinoma) and FTC-133 (human follicular thyroid carcinoma) cancer cell lines. Further studies such as AO-EB (acridine orange-ethidium bromide), nuclear staining and flow cytometry analyses on cell death mechanism signified that the cytotoxicity was associated with apoptosis in thyroid cancer cells. GEM-NPs show excellent biocompatibility compared to GEM. The present study explained that GEM-NPs as a safe and hopeful strategy for chemotherapeutics of thyroid cancer therapy and deserve for further clinical evaluations.

## 1. Introduction

Thyroid cancer is one of the usual endocrine malignancy cells with its occurrence steadily enhancing over the last couple of the years (Mahmoudian-Sani et al., 2019; McGonagle & Nucera, 2019; Simões-Pereira et al., 2019). Thyroid malignancy is commonly derived from stem of the thyroid follicular cells from epithelial, which can be additionally divided into the well distinguished malignancy cells, poorly discriminated malignance, and anaplastic malignance tumor cells rendering to the cancer behaviors. A distinguished thyroid malignancy cell is presently treated via thyroidectomy, succeeding radioactive iodine (RAI) treatment and long-standing thyroid triggering suppression of hormone (Bi et al., 2019; Corrigan et al., 2019; Ljubas et al., 2019; Pan et al., 2019). Although a majority of thyroid cancers have a favorable prognosis, there are scarcely any effective therapeutic approaches for metastatic thyroid cancers (Galofré et al., 2014; Kim et al., 2018; Ishihara et al., 2019; Janz et al., 2019; Wang et al., 2019). Traditional treatment strategies are usually not effective for those patients with nasty local invasion or distant metastasis, and patients harboring distant metastasis frequently deteriorate, with a nearly 50% 5-year survival rate. In addition, if dedifferentiation occurs, thyroid cancer patients will assume more risks of neoplasm metastasis and lose RAI avidity which is always related to a poor prognosis. Since, it is urgent to search a new approach to prevent the metastasis thyroid cancer patients (Chavez et al., 2018; Garcia et al., 2019; Shelly et al., 2019; Yamazaki et al., 2019).

Gemcitabine (GEM) and its derivatives of deoxycytosine display the excellent chemotherapy available for the treatment of all types of cancer cells in the pre-clinical use. In metastasis thyroid cancer, GEM is combined to reproduce the DNA terminal chain elongations, eventually triggering the apoptosis of the tumor cells (Awasthi et al., 2012; Qin et al., 2019; Saini et al., 2020). Although these GEM analogues are applied for the therapy of thyroid patients, the treatment outcomes continue high unsatisfactory results, increasing the median of survival of thyroid patient only month (Unnam et al., 2019). The pre-clinical performance of the GEM is extensively compromised via essential obstacles including the blood flow rate of half-lifer period, quick deactivations and defecations, establishment of the drug resistances, and potentially serves as a side effect. Consequently, there is an essential motivation for the increased net easy but efficiency methods to completely attach GEM, which can widen the efficacy of this drug type for the managements of demoralizing the thyroid cancer (Zhang et al., 2013; Delplace et al., 2014; Kim et al., 2015; Parker et al., 2016).

Drug-delivery nanosystems capable of modulating the physicochemical properties and the *in vivo* performance of given chemotherapeutic drugs are emerging for the treatment of various diseases. Also, GEM having a hydrophilic molecules and thus incompatible with numerous polymer nanoparticles are extensively utilized in the exact biological drug delivery (De Sanctis et al., 2019; Ogunwobi & Kumar, 2019; Yajima & Akise, 2019). Hence, the biological processes to initiate the compositions with hydrophilic GEM molecules with polymeric frame works characterize the considerable challenge. This shows the problems and to enhance the stability of the nanoparticles and bioavailability of the GEM thyroid cancer treatment, by using different methods (Liu et al., 2019; Sandblom et al., 2019; Akhavan et al., 2020). Recently, GEM and its prodrugs incorporated with PEG_2000_ micelles for the excellent antitumor agents are reported by Kwon and coworkers. More recently, Cattel and research groups reported the injectable nanomedicines using GEM and its analogues show greater solubility and bioavailability (Boloix et al., 2019; Kaur et al., 2019; Oh et al., 2019). The GEM amino groups acylation did not impair the *in vitro* cytotoxic potential of small molecule prodrugs associated with lone GEM molecules (Ahn et al., 2019; Cao et al., 2019; Sibio et al., 2019; Suzuki et al., 2019; Yao et al., 2019).

In addition, after systematic managements, unpreferred adaptation of GEM to inactive uracil metabolite analogues can be substantially inhibited since decreased catabolic effects of cytidine are abundant in the plasma membrane (Bunyatov et al., 2019; Iijima et al., 2020; Zhao et al., 2020). Therefore, the GEM encapsulation prodrug has been displayed to eliminate the drug deactivations and enhance the drug loading efficiency by misusing the improved permeability and retention effects (He et al., 2019; Thapi et al., 2019; Yan et al., 2019). In this work, we described easy and greater effect methods that actively conjugate with the drug reconstitution nano-assembly and tumor targeting agents of GEM therapies. To actively target the goal, LA is an essential fatty acid, conjugate with amino moiety of the GEM analogue through amide formation. Fruitfully, we found the GEM prodrugs increasing the self-assembly nanoparticles in the water solutions. The self-assembly GEM prodrug examined the *in vitro* cytotoxicity of thyroid cancers cells and established the morphological changes and mechanistic investigations of thyroid cancer cells.

## 2. Experimental section

### 2.1. Preparation of GEM prodrug conjugated with LA

**Figure 1.**
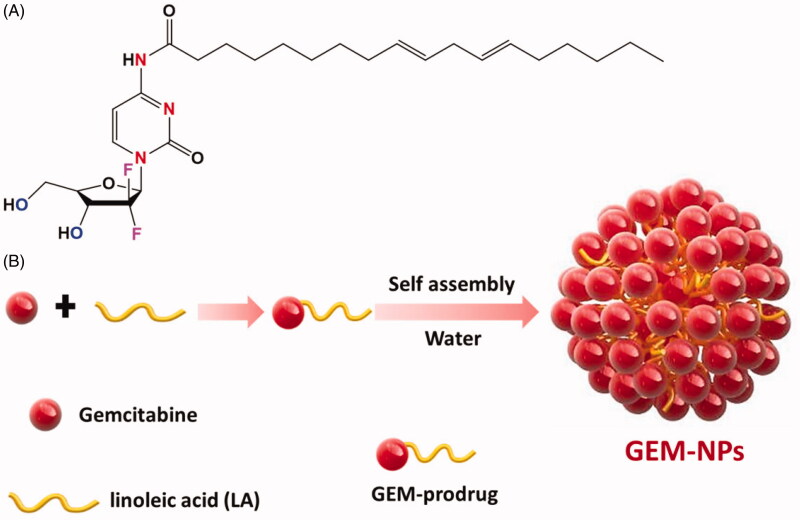
Schematic representation of thyroid cancer cell targeting GEM-NPs. (A) Structure of Gemcitabine (GEM). Graphic design of the preparation process of GEM prodrug formations and self-assembly of GEM-NPs in water showing potential chemotherapy effect.

GEM solution (50 mg, 0.19 mmol) and LA (53 mg, 0.19 mmol) having 1.5 mL of DMF was mixed into DIEA (97 mg, 7.2 mmol). The reaction mixture of the solution was allowed to stir at room temperature for overnight and then evaporated to remove DMF solution. The residue of the crude samples was immersed into the DCM. The organic layer was washed with 5% citric acid, NaCl, and aqueous NaHCO_3_. Later, the DCM layer was vacuum dried with Na_2_SO_4_ solutions. The detailed synthetic procedures are depicted in Figure 1.

### 2.2. Characterization

The structure and morphology of GEM-NPs were characterized by different techniques including *z*-potential and dynamic light scattering (DLS). Transmission electron microscopy (TEM) and high-resolution TEM (HRTEM) were conducted on an FEI Tecnai F20 transmission electron microscope with an accelerating voltage of 200 kV.

### 2.3. Cell culture

Thyroid cancer cell lines B-CPAP and FTC-133 have been collected from the Department of Thoracic Surgery, the First Hospital of Jilin University, PR China. B-CPAP cells are cultured in RPMI 1640 medium (Cell Signaling, Shanghai, China) and FTC-133 cancer cells are cultured in DMEM medium complemented by 10% fetal bovine serum (Cell Signaling, Shanghai, China), penicillin and 100 μg/mL streptomycin as antibiotics (Cell Signaling, Shanghai, China) in 96-well grown which were incubated under a humid atmosphere of 5% CO_2_ at 37 °C.

### 2.4. *In vitro* cytotoxicity of CCK-8 assay

A CCK-8 assay was conducted according to previously reported procedures (Akhter et al., 2020; Soni et al., 2020). It is used to determine the *in vitro* cytotoxicity of GEM and GEM-NPs. B-CPAP and FTC-133 cells were seeded into 96-well plates (4000–5000 cells in each well) and were cultured at 37 °C for 24 h. Then, the cells were subjected to constant dilution of GEM and GEM-NPs. Additionally, the result of nucleoside transporter inhibitors on the cytotoxicity of the GEM free was assessed. After incubation at 37 °C for 24 h, 10 μL of the CCK-8 solution was added to each well, and the absorbance at 520 nm was examined 2 h later by a microplate reader (Multiskan FC, Thermo Scientific, Waltham, MA). The percentage of cell inhibition by GEM and GEM-NPs was calculated and plotted in the graph by using GraphPad Prism Software (GraphPad Software, La Jolla, CA) (Ye et al., 2016; Tian et al., 2017; Cai et al., 2019). The percentage of cell viability was measured and cytotoxicity was shown as IC_50_ and the data shown represent the average of three independent experiments. The cell viability (%) was examined using the following formula:

$$\%\;\text{of}\;\text{cell}\;\text{viability}={\;\text{OD}}_{\text{treated}}/{\text{OD}}_{\text{control}}\times 100$$

### 2.5. Cellular uptake

The cellular uptake experiments are examined according to the manufacturer’s protocols. In six-well plate, 1 × 10^4^ B-CPAP and FTC-133 thyroid cancer cell lines were imagined by LCSM (Fluoview FV 1000, Olympus, Tokyo, Japan). GEM-NP was labeled with Dil (1,1′-dioctadecyl-3,3,3′,3′-tetramethylindocarbocyanine) for the examinations. In this experiment, cell lysosome was stained with Lysotracker Green DND-26 (Molecular Probe, Eugene, OR). Then, the nuclei were stained with DAPI. Subsequently, the coverslips were placed onto the glass microscope slides, and the cellular localization of GEM-NPs was pictured underneath LCSM.

### 2.6. *In vitro* drug release from the GEM and GEM-NPs

The release profiles of total GEM and GEM-NPs were monitored by dialysis using a membrane (Spectrum Laboratories, San Francisco, CA, molecular weight cut off 14 kDa). Briefly, 10 mL of GEM-NPs (0.1 mg/mL GEM or GEM-NPs equivalent concentration) were dialyzed against 20 mL of phosphate buffer solutions (PBS, pH 7.4, 0.2% Tween 80) at 37 °C. By continuous gentle stirring in an orbital shaker (100 rpm) at 37 °C, the releasing medium was collected and an equal volume of fresh medium was supplemented (You et al., 2012; Deng et al., 2015; Duo et al., 2017).

### 2.7. Dual (AO-EB) staining

Apoptotic morphological changes upon treatment with IC_50_ concentration of GEM and GEM-NPs against B-CPAP and FTC-133 cancer cell lines were measured by using acridine orange and ethidium bromide (AO/EB) and Hoechst-33344 staining. After staining, the cells were visualized under a fluorescence microscope (Accu Scope EXI-310, Commack, NY) at ×20 magnification (Mohamed Subarkhan et al., 2016a, 2016b, 2019; Subarkhan & Ramesh, 2016; Mohamed Kasim et al., 2018).

### 2.8. Flow cytometry/Annexin V-PI staining

The flow cytometry analysis with the fluorescein isothiocyanate (FITC) Annexin V Apoptosis Detection Kit (Multi Sciences, Hangzhou, China) was used to determine the B-CPAP and FTC-133 cancer cell lines apoptotic ratio. The cells were composed by trypsinization, and washed twice and resuspended in 500 μL 1 × binding buffer with 5 μL of FITC Annexin V and 10 μL of PI. After 15 min, the samples were subjected to analysis by flow cytometry. The outcomes were analyzed with the BD FACS Calibur™ system (Mohan et al., 2018).

### 2.9. Hemolysis of GEM and GEM-NPs

Freshly collected human blood samples were collected from the Department of General Practice, Zhumadian City Central Hospital, Zhumadian 46300, China, and it was permitted by the Ethical Committee of the Department of General Practice, Zhumadian City Central Hospital, Zhumadian, China. We have conducted hemolysis according to the previously reported procedures. The blood was centrifuged and the supernatant solutions were extracted and washed with cold PBS for three times and the blood was fully eliminated and the HRBCs are obtained. Later, the HRBC solutions (0.1) were diluted with cold PBS. The solution was moved to the 5 mL tubes with 0.9 mL of DD-water added and it was used as a positive control. Further, 0.9 mL was used as a negative control. Furthermore, this PBS contains solutions of GEM and GEM-NPs (5–30 μg/mL), respectively. Later, this mixture was incubated into 3 h, continued by centrifugation and the absorbance was calculated by UV-spectrometer using the general formula. % Hemolysis = (*A*_s_ – *A*_n_)/(*A*_p_ – *A*_n_) × 100%, where, *A*_s_, *A*_n_, and *A*_p_ are the absorbances of the sample, the negative control, and the positive control, respectively (Tramer et al., 2012; Pham et al., 2014; Chang et al., 2015).

## 3. Results and discussion

### 3.1. Synthesis and structural illustration of GEM

The GEM and GEM-NPs were prepared with some alteration in compliance with previous methods. Figure 1(B) displays the overall synthetic process. GEM analogues conjugate with various functional groups at the N-terminal ends to display the enhanced stability in the cellular plasma due to the deamination target. Further, the hydrophilic promoter of LA is utilized for the formation of amphiphilic prodrug. In this work, we designed and developed the GEM analogues to the LA functional groups to make a GEM-LA prodrug through the amide group (Figure 1). The conjugations were performed via coupling of GEM into LA under the formation of PyBOP, and the final GEM-LA prodrug was purified using TLC and column chromatographic methods with the excellent yields (75.6%). The amphiphilic nature of the prodrugs makes nanoassemblies to synthesize in the water through self-assembly methods without adding any surfactants. Additionally, the organic phases having GEM-LA prodrug was immersed in the DMSO solution into the DD-H_2_O formed water suspension. More interestingly, the extra removal of the organic solvents was done via dialyzation with DD-H_2_O which produced a nanosuspension rather than the prodrug precipitations. Further, the TEM examinations were performed to evaluate the morphology of the self-assembled GEM-NPs. The outcomes of the self-assembled GEM-NPs displayed the well-organized shape with spherical structure with ∼79.2 ± 9.25 nm. Further, the hydrodynamic parameter of the self-assembled GEM-NPs was 102.5 ± 1.25 nm with less PDI, as demonstrated via DLS methods. The excellent outcomes suggested the formation of the self-assembled GEM-NPs.

### 3.2. *In vitro* GEM release from the GEM-NPs

**Figure 2.**
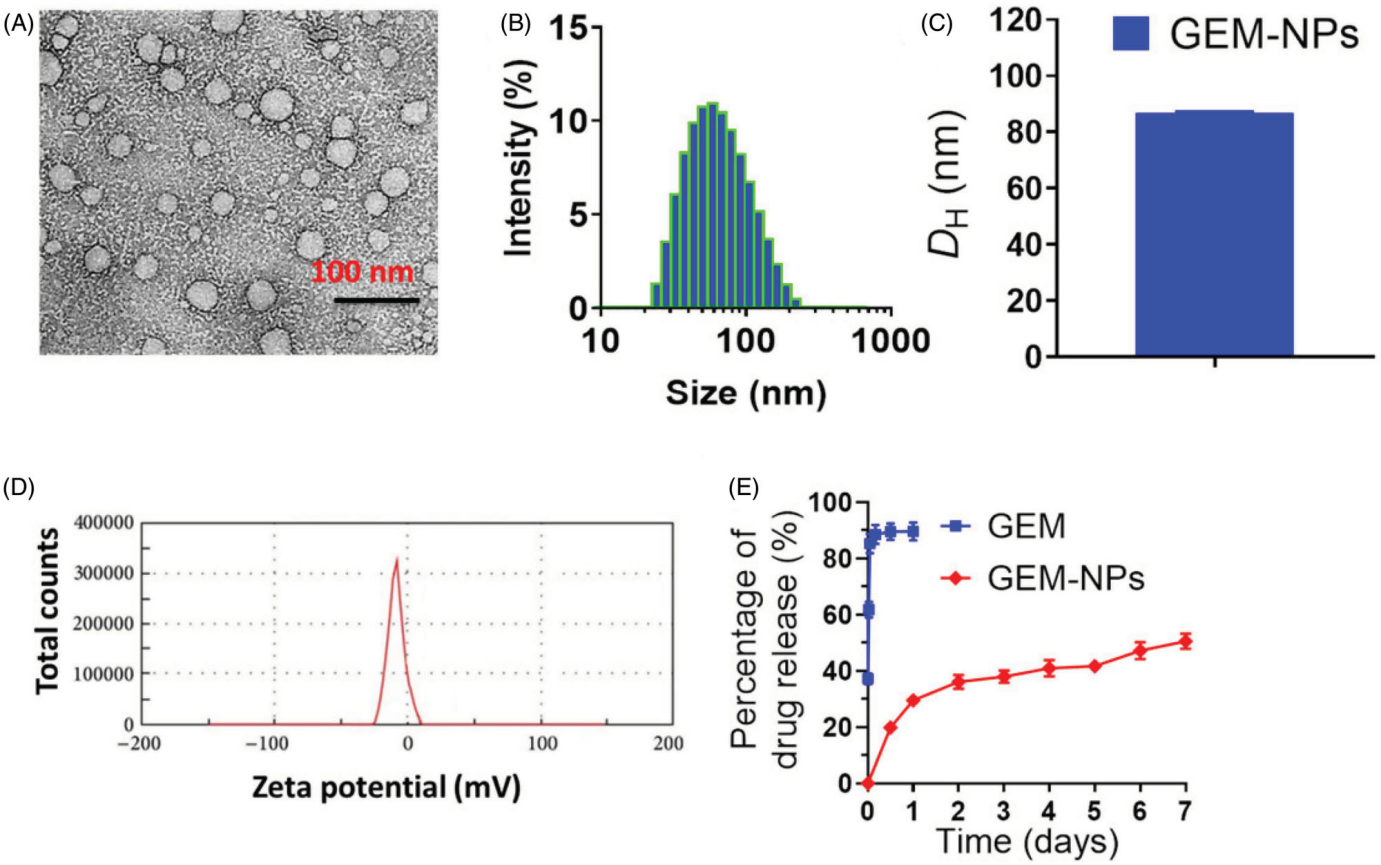
Characterization of GEM-NPs. (A) TEM image of GEM-NPs. Scale bar 100 nm. (B) Hydrodynamic parameter of GEM-NPs. (C) Diagram of hydrodynamic parameter of GEM-NPs. (D) Zeta potential examined via DLS analysis. (E) The solution containing GEM-NPs was dialyzed counter to PBS (pH 7.4) at 37 °C. *In vitro* drug release of GEM prodrug from GEM-NPs.

As a typical nucleoside analogue, the major deficiencies of GEM as an anticancer drug include short half-life in plasma and rapid deactivation by cytidine deaminase. We established the self-assembly GEM-NPs could aid as pool to stably constrain the medications and obstruct medications release through general blood circulation, thus decelerating GEM authorization from the human body. The releasing profiles of GEM-NPs are examined via dialyzing against PBS at room temperature. As depicted in Figure 2(E), GEM free was quickly liberated from the GEM-NPs and plateaued at 90.2 ± 2.9 release of drug after 25 h of incubations. In contrast, GEM-NPs exhibited sustained release kinetics. Our outcomes of *in vitro* drug lease displayed the slow inhibition release kinetics of GEM free from the GEM-NPs, which is helpful for extending the blood plasma half-life of GEM free and improving the drug conveyance into tumor cells.

### 3.3. Cellular uptake

**Figure 3.**
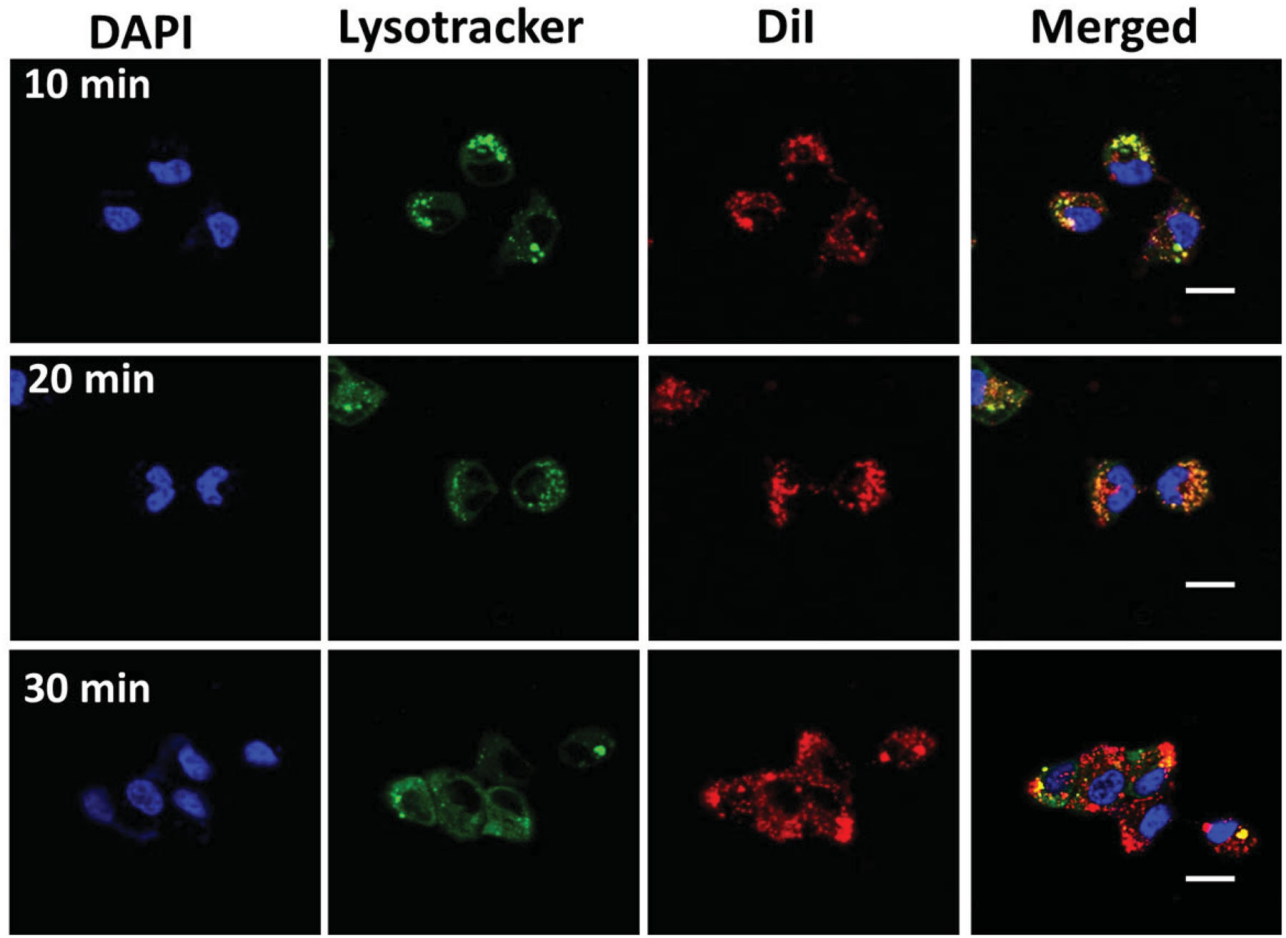
Subcellular localization of GEM-NPs with lysosomes in B-CPAP thyroid cancer cell lines at 10, 20, and 30 minutes incubation time. Scale bar = 20 μm.

We conjugated that the GEM-NPs enable the interface with B-CPAP and FTC-133 thyroid cancer cells and particles, hence improving the intracellular uptake into the thyroid cancer cell. To test this statement, examination of subcellular localization of GEM-NPs in the B-CPAP thyroid cancer cell lines was monitored by confocal laser scanning microscopy (LCSM) (Figure 3). The 20 nm concentrations of GEM-NPs were labeled with fluorescence marker with DiI (red color) for the investigations, on the contrast, the lysosome was stained with green LysoTracker-DND26, and the nuclei were stained with the DAPI (blue). The newly appeared yellow fluorescent was combined with LysoTracker-DND26 and DiI fluorescence in the B-CPAP thyroid cancer cell lines; therefore, GEM-NPs could be localized with lysosome after internalizations (Ahmad et al., 2020; Akhter & Amin, 2017; Akhter et al., 2018; Habban Akhter et al., 2018).

### 3.4. *In vitro* cytotoxicity

**Figure 4.**
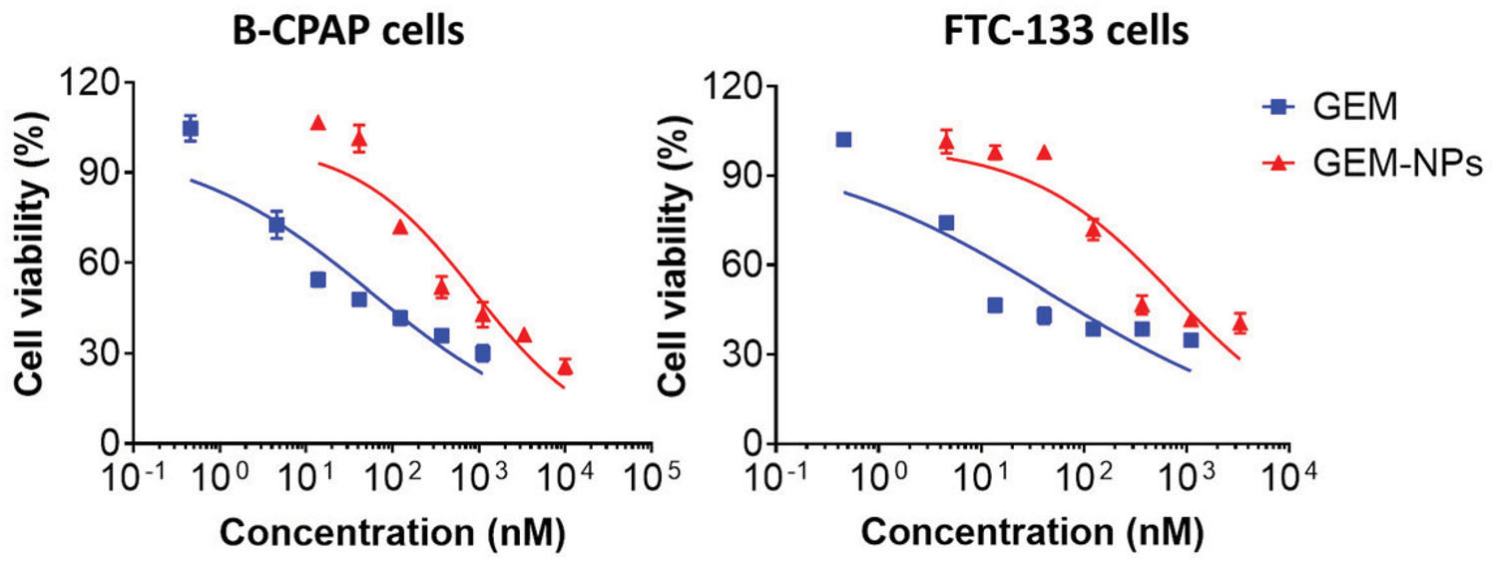
*In vitro* cytotoxicity of B-CPAP and FTC-133 thyroid cancer cell lines.

After successful synthesis of GEM-NPs, we performed the CCK-8 (cell counting kit-8) examinations to evaluate the cytotoxicity of the GEM and GEM-NPs to thyroid cancer cell lines, with B-CPAP and FTC-133 cancer cells. Next, on treatment with the medications for 24 h, the viability of the cells was monitored, and the half inhibitory concentration (IC_50_) was obtained from the dose-dependent curve (Figure 4). Encouragingly, compared with the free drug, prodrug-assembled nanoparticles showed substantially enhanced cytotoxic activity in both tested cell lines. For instance, in B-CPAP cell lines, the IC_50_ was 138.40 ± 11.12 and 13.62 ± 0.97 for GEM and GEM-NPs, respectively. In FTC-133 cell lines, the IC_50_ was 62.63 ± 3.30 and 25.16 ± 2.80 for GEM and GEM-NPs, respectively. Further, using free LA–GEM conjugate to test the cytotoxicity, we confirmed that PUFAylation of GEM at the 4-(N)-position indeed did not impair the potency of GEM. After entering the cells, the amide bond could be hydrolyzed by intracellular amidases such as cathepsin B or cathepsin D, thereby regenerating active GEM. GEM is a hydrophilic molecule; thus, it is difficult to diffuse through the lipid bilayer of the cell membrane. Therefore, efficient cellular uptake requires membrane nucleoside transporter proteins.

### 3.5. Morphological assessment

**Figure 5.**
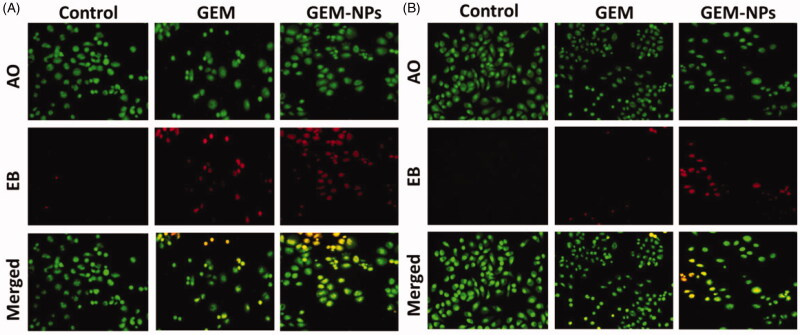
Dual AO/EB fluorescent staining of B-CPAP and FTC-133 thyroid cancer cell lines after treatment with GEM and GEM-NPs (IC_50_ concentration) for 24 h.

By using a fluorescent microscopic analysis of the AO-EB and Hoechst-33258 stained in B-CPAP and FTC-133 thyroid cancer cell lines, characteristic morphological changes induced by GEM and GEM-NPs were evaluated (Figure 5). The nanoparticle induces cell death via two pathways, such as apoptosis and necrosis, after treatment with their IC_50_ meditations at 24 hours. Ironically, GEM-NPs show a higher percentage of apoptotic modes of cell death than GEM free drug.

**Figure 6.**
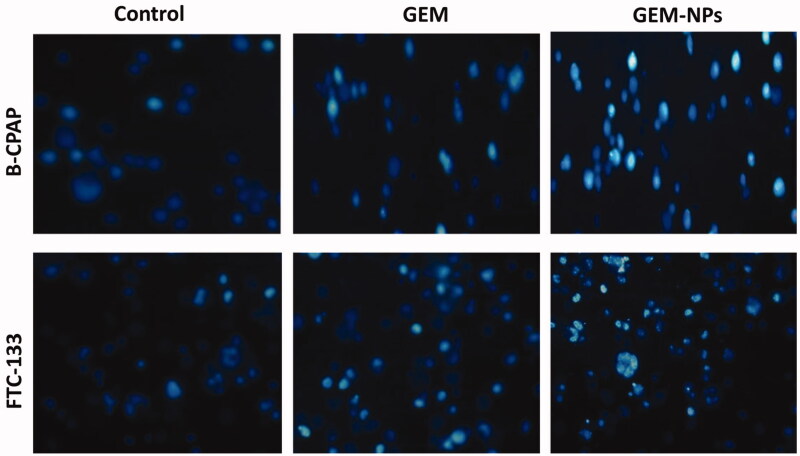
Nuclear staining of B-CPAP and FTC-133 thyroid cancer cell lines after treatment with GEM and GEM-NPs (IC_50_ concentration) for 24 h.

Once Hoechst-33258 staining is used, chromatin fragmentation, bi-and/or multinucleation, cytoplasmic vacuolation, nuclear swelling, cytoplasmic bleating, and late apoptosis suggestion of dot-like chromatin condensation are detected on morphological changes of thyroid cancer cells. The observed cytological changes are secreted into four sorts rendering to the emission of fluorescence and the morphological characteristics of chromatin condensation in the AO-EB stained nucleus: (i) live cells with consistently green fluorescent nucleus with extreme structure; (ii) early apoptotic cells (which still have intact membranes but have begun to undergo DNA fragmentation) with green fluorescent nodes, but peri-nuclear chromatin nuclear chromatin condensation is visible as bright green patches or fragments; (iii) late apoptotic cells with orange to red fluorescent nodes with condensed or fragmented chromatin; (iv) necrotic cells swollen to huge sizes, with uniformly orange to red fluorescent nucleus with no sign of chromatin fragmentation. All these morphological modifications suggest that the thyroid cancer cells undergo apoptosis mode of cell death over the treatment of 24 hours (Figure 6). It has been stated that the anticancer action of certain GEM and GEM-NPs depends on their interaction with different proteins and their modes of binding to duplex DNA. Whole binding of plasma proteins may result in drastic alterations or even loss of the biological properties of GEM and GEM-NPs.

### 3.6. Apoptosis by flow cytometry

**Figure 7.**
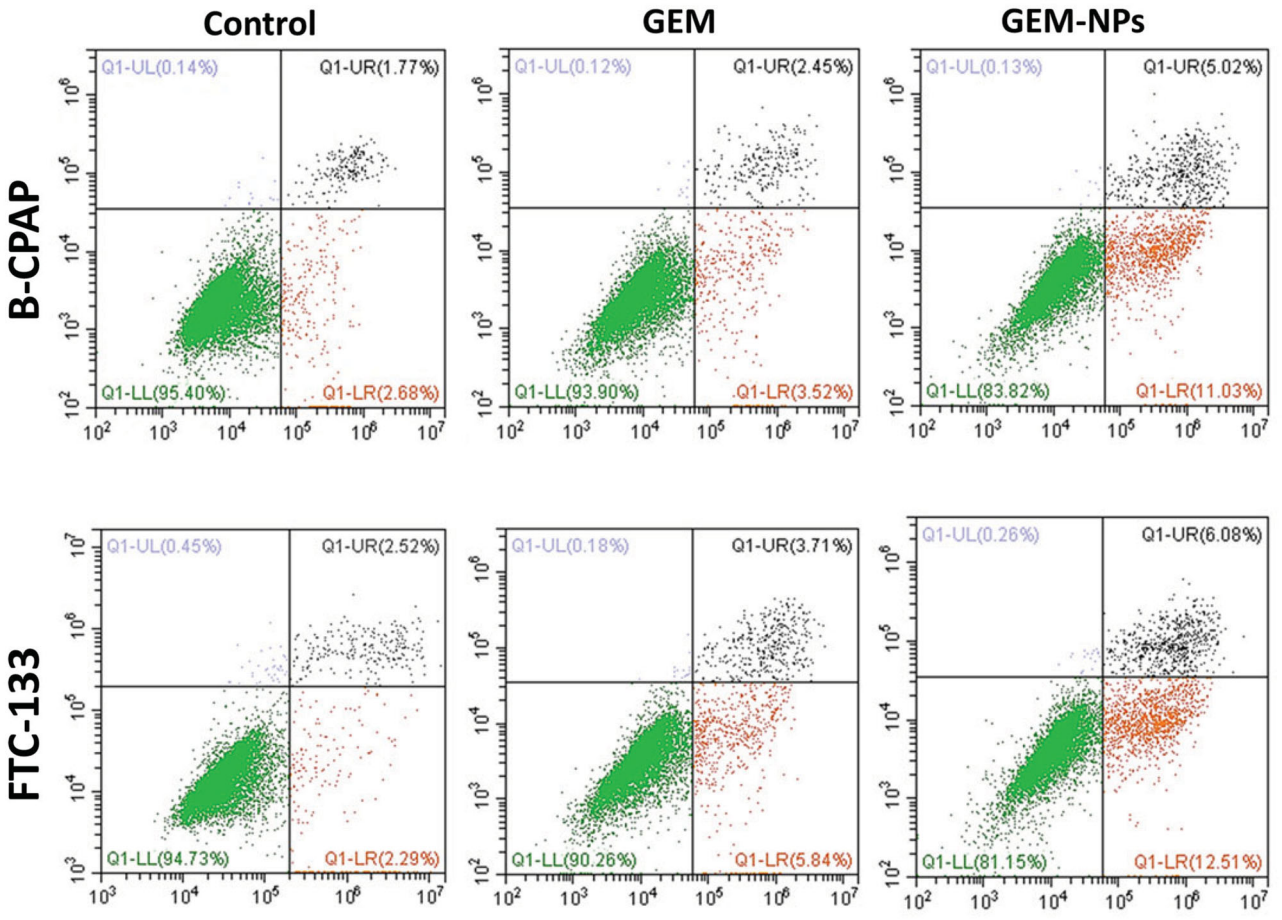
Apoptotic examination of B-CPAP and FTC-133 thyroid cancer cell lines using flow cytometry. The cells were treated with GEM and GEM-NPs (IC_50_ concentration) for 24 h, and stained with FITC annexin V/PI for flow cytometry investigation.

Apoptosis can be reckoned as an important impediment for a cell to become a developing cancer cell. Qualitative substantiation of apoptosis induction property of the complexes by AO-EB dual staining approach has prompted us to clarify and quantify the apoptosing cancer cells by flow cytometry using Alexa Fluor 488 Annexin V combined with propidium iodide (PI) staining assay in B-CPAP and FTC-133 thyroid cancer cell lines. Annexin V is a cell membrane impermeable protein having very high affinity toward phosphatidylserine (PS). PS is a cell cycle signaling phospholipid located inside the membrane of a healthy cell but is reverted to the outer membrane at the time of apoptosis. Hence, early and late apoptotic cells were identified as green images in the four quadrants diagram of flow cytometry by binding fluorescent conjugated Annexin V with exposed PS. PI is a DNA intercalating fluorescent dye also impermeable to the intact cell membrane but can penetrate through the damaged membrane of late apoptotic and early necrotic cells and binds to DNA fluorescent red. The cells at different stages, i.e. live cells, early apoptotic cells and late apoptotic cells were quantified in Annexin V–/PI–, Annexin V+/PI–, and Annexin V+/PI + quadrants respectively using fluorescence-activated cell sorting (FACS) methodology. GEM and GEM-NPs treated cancer cells exhibit different degrees of apoptosis. The late apoptotic cells are clearly indicated in Figure 7 along with the percentage of apoptotic cells. These results revealed that the complexes significantly induce apoptosis.

### 3.7. Hemolysis assay of GEM and GEM-NPs

**Figure 8.**
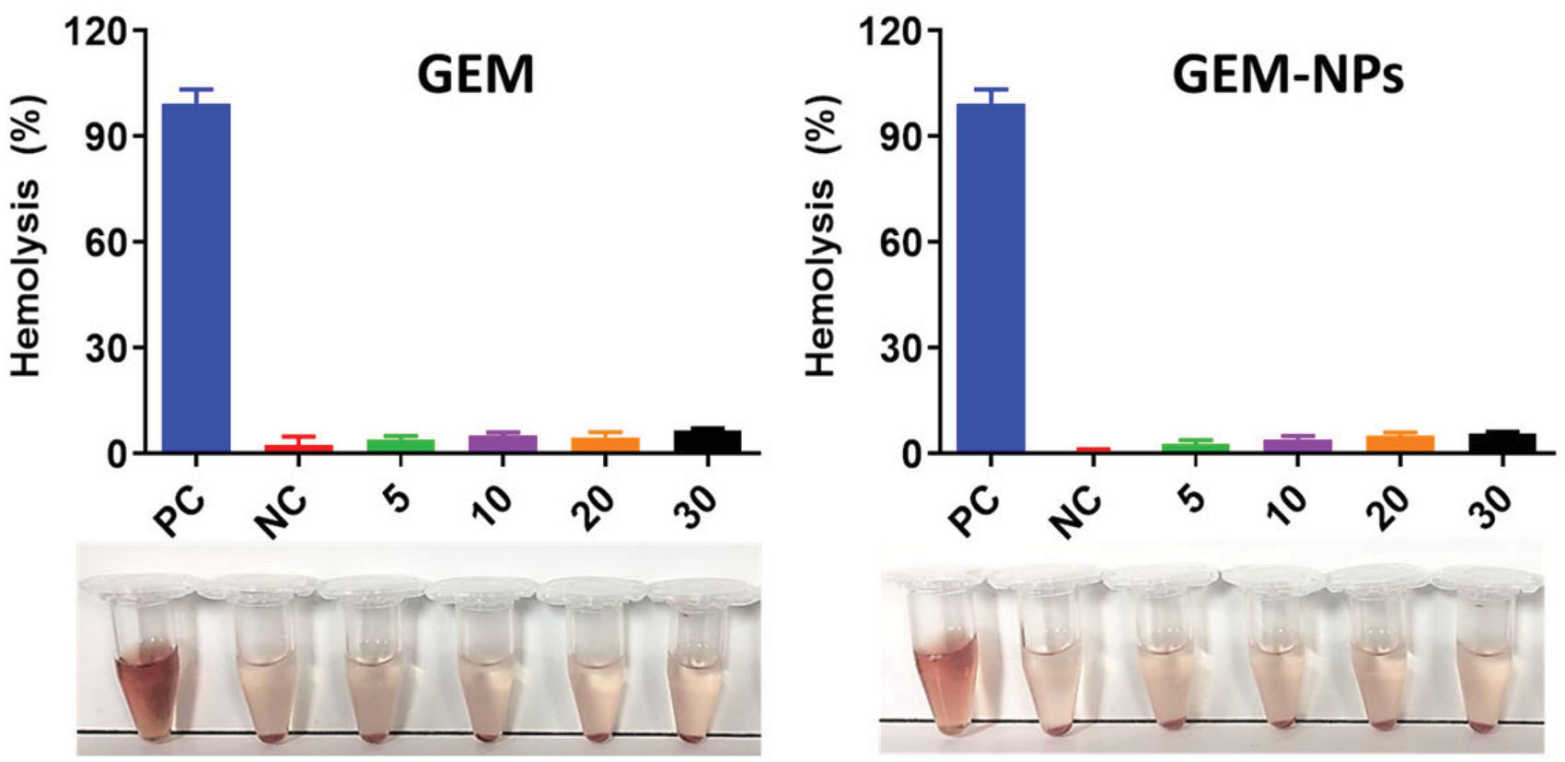
Hemolysis assay with different concentration of GEM and GEM-NPs. The result of hemolysis assay reveals that the insignificant hemolysis shows that it is extremely biocompatible for *in vivo* profiles.

The nanoformulations are predictable to interrelate with human red blood cells and cause the cell membrane damaging by hemolysis. In order to examine the human health of such adverse effects, *in vitro* biocompatibility assay was examined. The biocompatibility profiles of RBC caused by the nanoparticles were demonstrated by the different concentrations 5–30 µg/mL. Figure 8 displays the dose-dependent hemolytic effect which shows reducing toxicity of GEM and GEM-NPs. According to the results of GEM and GEM-NPs, we detected only insignificant hemolysis which shows that is extremely biocompatible for *in vivo* profiles.

## 4. Conclusions

In conclusion, we have successfully designed and demonstrated the hydrophilic and quick metabolized GEM prodrug to the biologically more efficient nanodrug. We point out the self-assembled GEM nanoparticles that show high DL, controlled release, and improved intracellular uptake to potentially overcome tumor cell accumulations through the effect of EPR. After positive synthesis, we have evaluated the MTT of GEM and GEM-NPs nanoparticles against B-CPAP and FTC-133 thyroid cancer cell lines. Further studies such as AO-EB (acridine orange-ethidium bromide), and flow cytometry analyses on cell death mechanism signified that the cytotoxicity was associated with apoptosis in thyroid cancer cells. Further, the morphological changes were observed using AO-EB and Hoechst-33258 staining. The results of GEM and GEM-NPs nanoparticles would invent the probable use in thyroid patients with improved chemotherapy and future clinical investigation.
